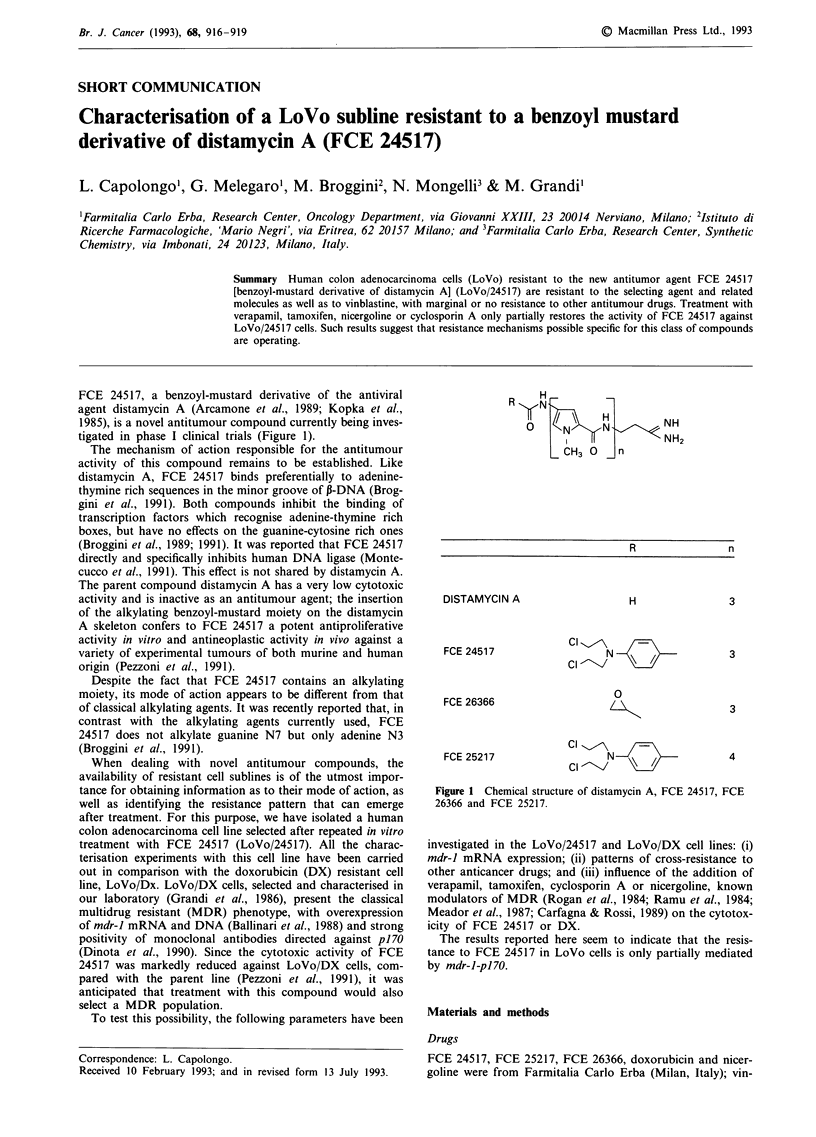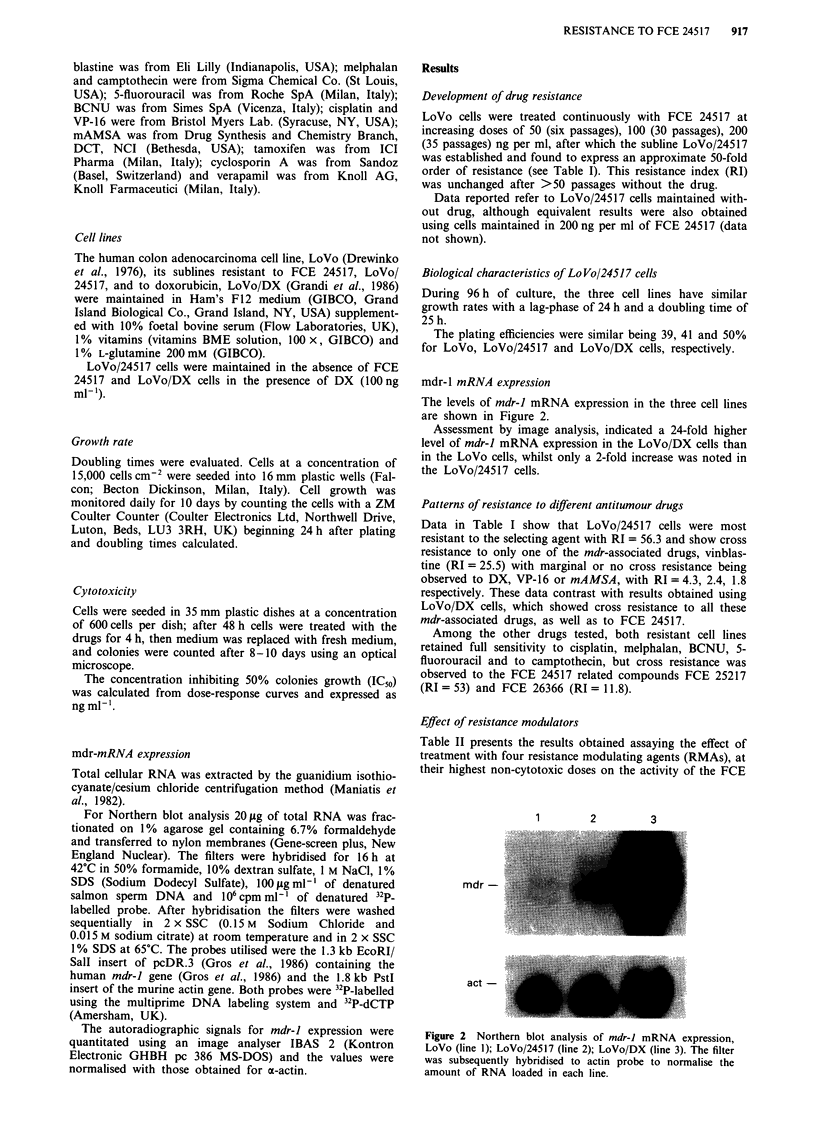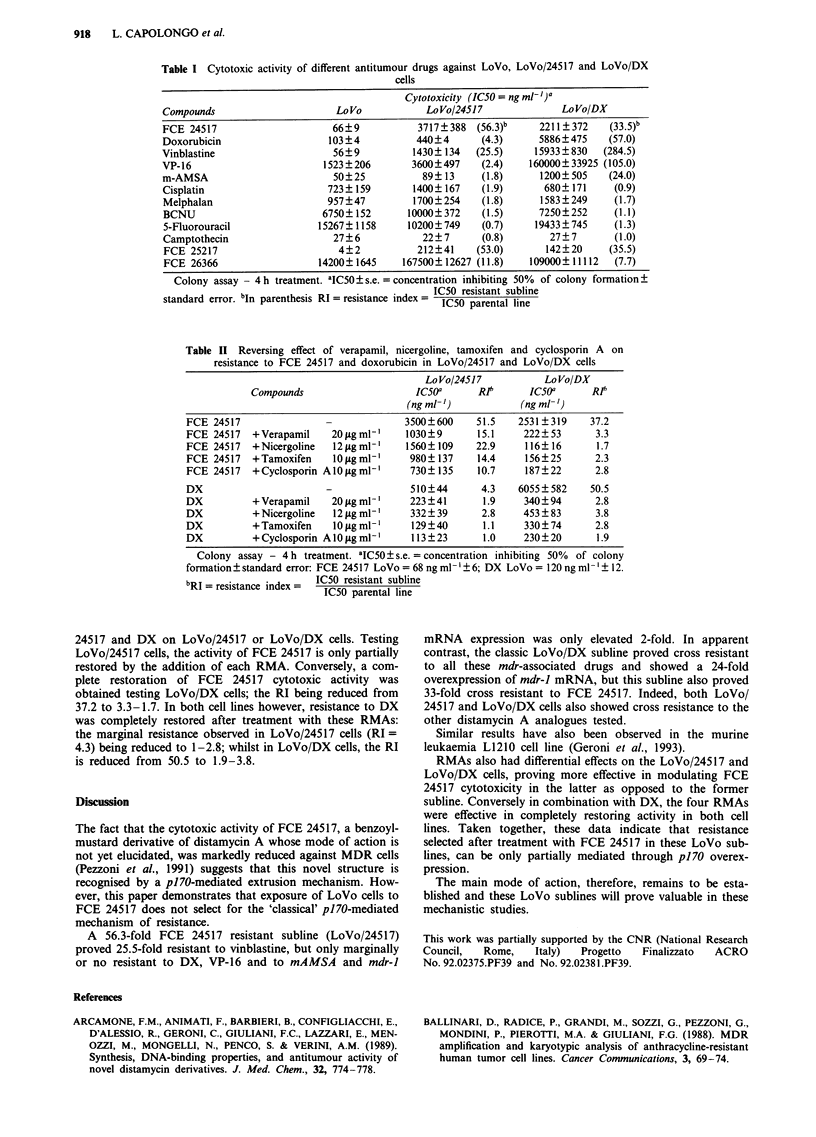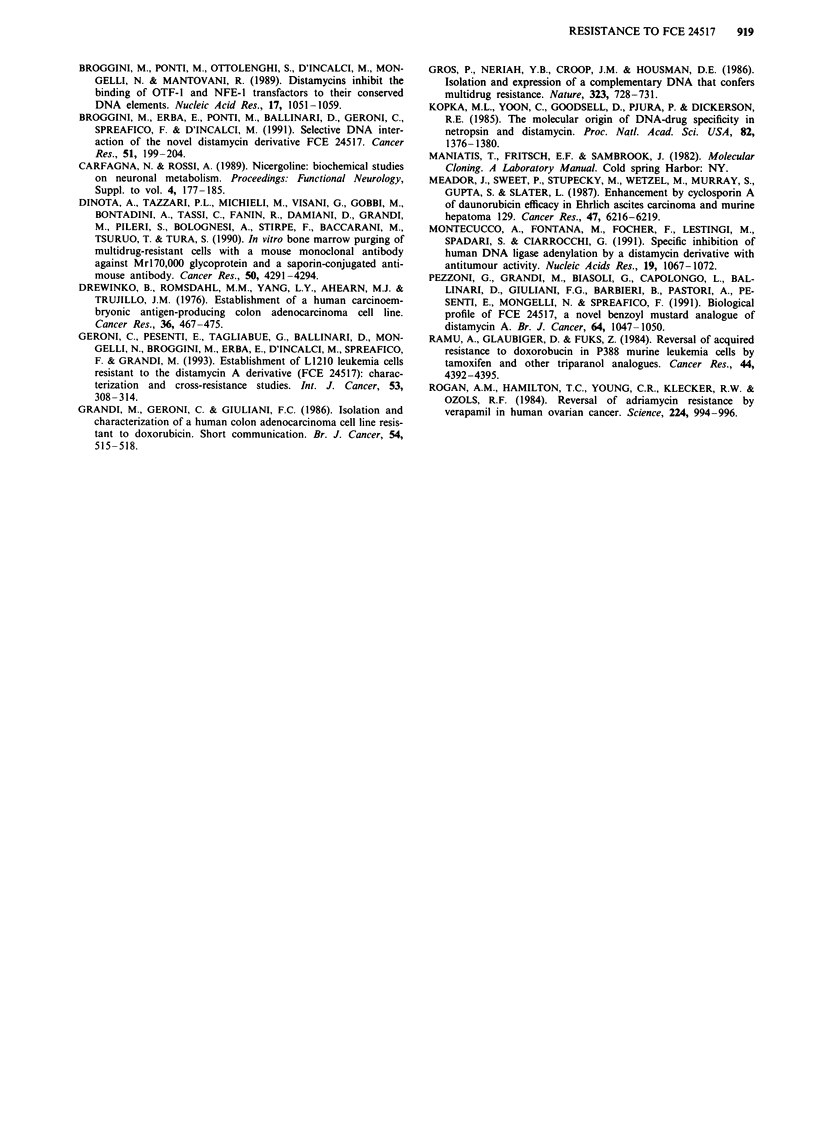# Characterisation of a LoVo subline resistant to a benzoyl mustard derivative of distamycin A (FCE 24517).

**DOI:** 10.1038/bjc.1993.454

**Published:** 1993-11

**Authors:** L. Capolongo, G. Melegaro, M. Broggini, N. Mongelli, M. Grandi

**Affiliations:** Farmitalia Carlo Erba, Research Center, Oncology Department, Nerviano, Milano, Italy.

## Abstract

**Images:**


*Br. J. Cancer* (1993), **68**, 916–919 © Macmillan Press Ltd., 1993

SHORT COMMUNICATION

### Characterisation of a LoVo subline resistant to a benzoyl mustard derivative of distamycin A (FCE 24517)

L. Capolongo[1], G. Melegaro[1], M. Broggini[2], N. Mongelli[3] & M. Grandi[1]

[1]*Farmitalia Carlo Erba, Research Center, Oncology Department, via Giovanni XXIII, 23 20014 Nerviano, Milano;* [2]*Istituto di Ricerche Farmacologiche, 'Mario Negri', via Eritrea, 62 20157 Milano; and* [3]*Farmitalia Carlo Erba, Research Center, Synthetic Chemistry, via Imbonati, 24 20123, Milano, Italy.*

**Summary** Human colon adenocarcinoma cells (LoVo) resistant to the new antitumor agent FCE 24517 [benzoyl-mustard derivative of distamycin A] (LoVo/24517) are resistant to the selecting agent and related molecules as well as to vinblastine, with marginal or no resistance to other antitumour drugs. Treatment with verapamil, tamoxifen, nicergoline or cyclosporin A only partially restores the activity of FCE 24517 against LoVo/24517 cells. Such results suggest that resistance mechanisms possible specific for this class of compounds are operating.

FCE 24517, a benzoyl-mustard derivative of the antiviral agent distamycin A (Arcamone *et al.*, 1989; Kopka *et al.*, 1985), is a novel antitumour compound currently being investigated in phase I clinical trials (Figure 1).

The mechanism of action responsible for the antitumour activity of this compound remains to be established. Like distamycin A, FCE 24517 binds preferentially to adenine-thymine rich sequences in the minor groove of β-DNA (Broggini *et al.*, 1991). Both compounds inhibit the binding of transcription factors which recognise adenine-thymine rich boxes, but have no effects on the guanine-cytosine rich ones (Broggini *et al.*, 1989; 1991). It was reported that FCE 24517 directly and specifically inhibits human DNA ligase (Montecucco *et al.*, 1991). This effect is not shared by distamycin A. The parent compound distamycin A has a very low cytotoxic activity and is inactive as an antitumour agent; the insertion of the alkylating benzoyl-mustard moiety on the distamycin A skeleton confers to FCE 24517 a potent antiproliferative activity *in vitro* and antineoplastic activity *in vivo* against a variety of experimental tumours of both murine and human origin (Pezzoni *et al.*, 1991).

Despite the fact that FCE 24517 contains an alkylating moiety, its mode of action appears to be different from that of classical alkylating agents. It was recently reported that, in contrast with the alkylating agents currently used, FCE 24517 does not alkylate guanine N7 but only adenine N3 (Broggini *et al.*, 1991).

When dealing with novel antitumour compounds, the availability of resistant cell sublines is of the utmost importance for obtaining information as to their mode of action, as well as identifying the resistance pattern that can emerge after treatment. For this purpose, we have isolated a human colon adenocarcinoma cell line selected after repeated *in vitro* treatment with FCE 24517 (LoVo/24517). All the characterisation experiments with this cell line have been carried out in comparison with the doxorubicin (DX) resistant cell line, LoVo/Dx. LoVo/DX cells, selected and characterised in our laboratory (Grandi *et al.*, 1986), present the classical multidrug resistant (MDR) phenotype, with overexpression of *mdr-1* mRNA and DNA (Ballinari *et al.*, 1988) and strong positivity of monoclonal antibodies directed against *p170* (Dinota *et al.*, 1990). Since the cytotoxic activity of FCE 24517 was markedly reduced against LoVo/DX cells, compared with the parent line (Pezzoni *et al.*, 1991), it was anticipated that treatment with this compound would also select a MDR population.

To test this possibility, the following parameters have been investigated in the LoVo/24517 and LoVo/DX cell lines: (i) *mdr-1* mRNA expression; (ii) patterns of cross-resistance to other anticancer drugs; and (iii) influence of the addition of verapamil, tamoxifen, cyclosporin A or nicergoline, known modulators of MDR (Rogan *et al.*, 1984; Ramu *et al.*, 1984; Meador *et al.*, 1987; Carfagna & Rossi, 1989) on the cytotoxicity of FCE 24517 or DX.

The results reported here seem to indicate that the resistance to FCE 24517 in LoVo cells is only partially mediated by *mdr-1-p170*.

**Figure 1** Chemical structure of distamycin A, FCE 24517, FCE 26366 and FCE 25217.

### Materials and methods

### *Drugs*

FCE 24517, FCE 25217, FCE 26366, doxorubicin and nicergoline were from Farmitalia Carlo Erba (Milan, Italy); vin-

Correspondence: L. Capolongo.
Received 10 February 1993; and in revised form 13 July 1993.

blastine was from Eli Lilly (Indianapolis, USA); melphalan and camptothecin were from Sigma Chemical Co. (St Louis, USA); 5-fluorouracil was from Roche SpA (Milan, Italy); BCNU was from Simes SpA (Vicenza, Italy); cisplatin and VP-16 were from Bristol Myers Lab. (Syracuse, NY, USA); mAMSA was from Drug Synthesis and Chemistry Branch, DCT, NCI (Bethesda, USA); tamoxifen was from ICI Pharma (Milan, Italy); cyclosporin A was from Sandoz (Basel, Switzerland) and verapamil was from Knoll AG, Knoll Farmaceutici (Milan, Italy).

### *Cell lines*

The human colon adenocarcinoma cell line, LoVo (Drewinko *et al.*, 1976), its sublines resistant to FCE 24517, LoVo/24517, and to doxorubicin, LoVo/DX (Grandi *et al.*, 1986) were maintained in Ham's F12 medium (GIBCO, Grand Island Biological Co., Grand Island, NY, USA) supplemented with 10% foetal bovine serum (Flow Laboratories, UK), 1% vitamins (vitamins BME solution, 100 ×, GIBCO) and 1% L-glutamine 200 mM (GIBCO).

LoVo/24517 cells were maintained in the absence of FCE 24517 and LoVo/DX cells in the presence of DX (100 ng $ml^{-1}$).

### *Growth rate*

Doubling times were evaluated. Cells at a concentration of 15,000 cells $cm^{-2}$ were seeded into 16 mm plastic wells (Falcon; Becton Dickinson, Milan, Italy). Cell growth was monitored daily for 10 days by counting the cells with a ZM Coulter Counter (Coulter Electronics Ltd, Northwell Drive, Luton, Beds, LU3 3RH, UK) beginning 24 h after plating and doubling times calculated.

### *Cytotoxicity*

Cells were seeded in 35 mm plastic dishes at a concentration of 600 cells per dish; after 48 h cells were treated with the drugs for 4 h, then medium was replaced with fresh medium, and colonies were counted after 8–10 days using an optical microscope.

The concentration inhibiting 50% colonies growth ($IC_{50}$) was calculated from dose-response curves and expressed as ng $ml^{-1}$.

### mdr-*mRNA expression*

Total cellular RNA was extracted by the guanidium isothiocyanate/cesium chloride centrifugation method (Maniatis *et al.*, 1982).

For Northern blot analysis 20 µg of total RNA was fractionated on 1% agarose gel containing 6.7% formaldehyde and transferred to nylon membranes (Gene-screen plus, New England Nuclear). The filters were hybridised for 16 h at 42°C in 50% formamide, 10% dextran sulfate, 1 M NaCl, 1% SDS (Sodium Dodecyl Sulfate), 100 µg $ml^{-1}$ of denatured salmon sperm DNA and $10^6$ cpm $ml^{-1}$ of denatured $^{32}P$-labelled probe. After hybridisation the filters were washed sequentially in 2 × SSC (0.15 M Sodium Chloride and 0.015 M sodium citrate) at room temperature and in 2 × SSC 1% SDS at 65°C. The probes utilised were the 1.3 kb EcoRI/SalI insert of pcDR.3 (Gros *et al.*, 1986) containing the human *mdr-1* gene (Gros *et al.*, 1986) and the 1.8 kb PstI insert of the murine actin gene. Both probes were $^{32}P$-labelled using the multiprime DNA labeling system and $^{32}P$-dCTP (Amersham, UK).

The autoradiographic signals for *mdr-1* expression were quantitated using an image analyser IBAS 2 (Kontron Electronic GHBH pc 386 MS-DOS) and the values were normalised with those obtained for α-actin.

### Results

### *Development of drug resistance*

LoVo cells were treated continuously with FCE 24517 at increasing doses of 50 (six passages), 100 (30 passages), 200 (35 passages) ng per ml, after which the subline LoVo/24517 was established and found to express an approximate 50-fold order of resistance (see Table I). This resistance index (RI) was unchanged after >50 passages without the drug.

Data reported refer to LoVo/24517 cells maintained without drug, although equivalent results were also obtained using cells maintained in 200 ng per ml of FCE 24517 (data not shown).

### *Biological characteristics of LoVo/24517 cells*

During 96 h of culture, the three cell lines have similar growth rates with a lag-phase of 24 h and a doubling time of 25 h.

The plating efficiencies were similar being 39, 41 and 50% for LoVo, LoVo/24517 and LoVo/DX cells, respectively.

### mdr-1 *mRNA expression*

The levels of *mdr-1* mRNA expression in the three cell lines are shown in Figure 2.

Assessment by image analysis, indicated a 24-fold higher level of *mdr-1* mRNA expression in the LoVo/DX cells than in the LoVo cells, whilst only a 2-fold increase was noted in the LoVo/24517 cells.

### *Patterns of resistance to different antitumour drugs*

Data in Table I show that LoVo/24517 cells were most resistant to the selecting agent with RI = 56.3 and show cross resistance to only one of the *mdr*-associated drugs, vinblastine (RI = 25.5) with marginal or no cross resistance being observed to DX, VP-16 or *mAMSA*, with RI = 4.3, 2.4, 1.8 respectively. These data contrast with results obtained using LoVo/DX cells, which showed cross resistance to all these *mdr*-associated drugs, as well as to FCE 24517.

Among the other drugs tested, both resistant cell lines retained full sensitivity to cisplatin, melphalan, BCNU, 5-fluorouracil and to camptothecin, but cross resistance was observed to the FCE 24517 related compounds FCE 25217 (RI = 53) and FCE 26366 (RI = 11.8).

### *Effect of resistance modulators*

Table II presents the results obtained assaying the effect of treatment with four resistance modulating agents (RMAs), at their highest non-cytotoxic doses on the activity of the FCE

**Figure 2** Northern blot analysis of *mdr-1* mRNA expression, LoVo (line 1); LoVo/24517 (line 2); LoVo/DX (line 3). The filter was subsequently hybridised to actin probe to normalise the amount of RNA loaded in each line.

**Table I** Cytotoxic activity of different antitumour drugs against LoVo, LoVo/24517 and LoVo/DX cells

| | Cytotoxicity ($IC50 = ng\ ml^{-1}$)[a] | | | | |
|---|---|---|---|---|---|
| *Compounds* | *LoVo* | *LoVo/24517* | | *LoVo/DX* | |
| FCE 24517 | 66±9 | 3717±388 | (56.3)[b] | 2211±372 | (33.5)[b] |
| Doxorubicin | 103±4 | 440±4 | (4.3) | 5886±475 | (57.0) |
| Vinblastine | 56±9 | 1430±134 | (25.5) | 15933±830 | (284.5) |
| VP-16 | 1523±206 | 3600±497 | (2.4) | 160000±33925 | (105.0) |
| m-AMSA | 50±25 | 89±13 | (1.8) | 1200±505 | (24.0) |
| Cisplatin | 723±159 | 1400±167 | (1.9) | 680±171 | (0.9) |
| Melphalan | 957±47 | 1700±254 | (1.8) | 1583±249 | (1.7) |
| BCNU | 6750±152 | 10000±372 | (1.5) | 7250±252 | (1.1) |
| 5-Fluorouracil | 15267±1158 | 10200±749 | (0.7) | 19433±745 | (1.3) |
| Camptothecin | 27±6 | 22±7 | (0.8) | 27±7 | (1.0) |
| FCE 25217 | 4±2 | 212±41 | (53.0) | 142±20 | (35.5) |
| FCE 26366 | 14200±1645 | 167500±12627 | (11.8) | 109000±11112 | (7.7) |

Colony assay - 4 h treatment. [a]IC50±s.e. = concentration inhibiting 50% of colony formation± standard error. [b]In parenthesis RI = resistance index = $\frac{\text{IC50 resistant subline}}{\text{IC50 parental line}}$

**Table II** Reversing effect of verapamil, nicergoline, tamoxifen and cyclosporin A on resistance to FCE 24517 and doxorubicin in LoVo/24517 and LoVo/DX cells

| | *Compounds* | | *LoVo/24517* IC50[a] ($ng\ ml^{-1}$) | RI[b] | *LoVo/DX* IC50[a] ($ng\ ml^{-1}$) | RI[b] |
|---|---|---|---|---|---|---|
| FCE 24517 | – | | 3500±600 | 51.5 | 2531±319 | 37.2 |
| FCE 24517 | +Verapamil | 20 μg ml$^{-1}$ | 1030±9 | 15.1 | 222±53 | 3.3 |
| FCE 24517 | +Nicergoline | 12 μg ml$^{-1}$ | 1560±109 | 22.9 | 116±16 | 1.7 |
| FCE 24517 | +Tamoxifen | 10 μg ml$^{-1}$ | 980±137 | 14.4 | 156±25 | 2.3 |
| FCE 24517 | +Cyclosporin A | 10 μg ml$^{-1}$ | 730±135 | 10.7 | 187±22 | 2.8 |
| DX | – | | 510±44 | 4.3 | 6055±582 | 50.5 |
| DX | +Verapamil | 20 μg ml$^{-1}$ | 223±41 | 1.9 | 340±94 | 2.8 |
| DX | +Nicergoline | 12 μg ml$^{-1}$ | 332±39 | 2.8 | 453±83 | 3.8 |
| DX | +Tamoxifen | 10 μg ml$^{-1}$ | 129±40 | 1.1 | 330±74 | 2.8 |
| DX | +Cyclosporin A | 10 μg ml$^{-1}$ | 113±23 | 1.0 | 230±20 | 1.9 |

Colony assay - 4 h treatment. [a]IC50±s.e. = concentration inhibiting 50% of colony formation±standard error: FCE 24517 LoVo = 68 ng ml$^{-1}$±6; DX LoVo = 120 ng ml$^{-1}$±12. [b]RI = resistance index = $\frac{\text{IC50 resistant subline}}{\text{IC50 parental line}}$

24517 and DX on LoVo/24517 or LoVo/DX cells. Testing LoVo/24517 cells, the activity of FCE 24517 is only partially restored by the addition of each RMA. Conversely, a complete restoration of FCE 24517 cytotoxic activity was obtained testing LoVo/DX cells; the RI being reduced from 37.2 to 3.3–1.7. In both cell lines however, resistance to DX was completely restored after treatment with these RMAs: the marginal resistance observed in LoVo/24517 cells (RI = 4.3) being reduced to 1–2.8; whilst in LoVo/DX cells, the RI is reduced from 50.5 to 1.9–3.8.

### Discussion

The fact that the cytotoxic activity of FCE 24517, a benzoyl-mustard derivative of distamycin A whose mode of action is not yet elucidated, was markedly reduced against MDR cells (Pezzoni *et al.*, 1991) suggests that this novel structure is recognised by a *p170*-mediated extrusion mechanism. However, this paper demonstrates that exposure of LoVo cells to FCE 24517 does not select for the 'classical' *p170*-mediated mechanism of resistance.

A 56.3-fold FCE 24517 resistant subline (LoVo/24517) proved 25.5-fold resistant to vinblastine, but only marginally or no resistant to DX, VP-16 and to *mAMSA* and *mdr-1* mRNA expression was only elevated 2-fold. In apparent contrast, the classic LoVo/DX subline proved cross resistant to all these *mdr*-associated drugs and showed a 24-fold overexpression of *mdr-1* mRNA, but this subline also proved 33-fold cross resistant to FCE 24517. Indeed, both LoVo/24517 and LoVo/DX cells also showed cross resistance to the other distamycin A analogues tested.

Similar results have also been observed in the murine leukaemia L1210 cell line (Geroni *et al.*, 1993).

RMAs also had differential effects on the LoVo/24517 and LoVo/DX cells, proving more effective in modulating FCE 24517 cytotoxicity in the latter as opposed to the former subline. Conversely in combination with DX, the four RMAs were effective in completely restoring activity in both cell lines. Taken together, these data indicate that resistance selected after treatment with FCE 24517 in these LoVo sublines, can be only partially mediated through *p170* overexpression.

The main mode of action, therefore, remains to be established and these LoVo sublines will prove valuable in these mechanistic studies.

This work was partially supported by the CNR (National Research Council, Rome, Italy) Progetto Finalizzato ACRO No. 92.02375.PF39 and No. 92.02381.PF39.

### References

ARCAMONE, F.M., ANIMATI, F., BARBIERI, B., CONFIGLIACCHI, E., D'ALESSIO, R., GERONI, C., GIULIANI, F.C., LAZZARI, E., MENOZZI, M., MONGELLI, N., PENCO, S. & VERINI, A.M. (1989). Synthesis, DNA-binding properties, and antitumour activity of novel distamycin derivatives. *J. Med. Chem.*, **32,** 774–778.

BALLINARI, D., RADICE, P., GRANDI, M., SOZZI, G., PEZZONI, G., MONDINI, P., PIEROTTI, M.A. & GIULIANI, F.G. (1988). MDR amplification and karyotypic analysis of anthracycline-resistant human tumor cell lines. *Cancer Communications*, **3,** 69–74.

BROGGINI, M., PONTI, M., OTTOLENGHI, S., D'INCALCI, M., MONGELLI, N. & MANTOVANI, R. (1989). Distamycins inhibit the binding of OTF-1 and NFE-1 transfactors to their conserved DNA elements. *Nucleic Acid Res.*, **17**, 1051–1059.

BROGGINI, M., ERBA, E., PONTI, M., BALLINARI, D., GERONI, C., SPREAFICO, F. & D'INCALCI, M. (1991). Selective DNA interaction of the novel distamycin derivative FCE 24517. *Cancer Res.*, **51**, 199–204.

CARFAGNA, N. & ROSSI, A. (1989). Nicergoline: biochemical studies on neuronal metabolism. *Proceedings: Functional Neurology*, Suppl. to vol. **4**, 177–185.

DINOTA, A., TAZZARI, P.L., MICHIELI, M., VISANI, G., GOBBI, M., BONTADINI, A., TASSI, C., FANIN, R., DAMIANI, D., GRANDI, M., PILERI, S., BOLOGNESI, A., STIRPE, F., BACCARANI, M., TSURUO, T. & TURA, S. (1990). *In vitro* bone marrow purging of multidrug-resistant cells with a mouse monoclonal antibody against Mr170,000 glycoprotein and a saporin-conjugated anti-mouse antibody. *Cancer Res.*, **50**, 4291–4294.

DREWINKO, B., ROMSDAHL, M.M., YANG, L.Y., AHEARN, M.J. & TRUJILLO, J.M. (1976). Establishment of a human carcinoembryonic antigen-producing colon adenocarcinoma cell line. *Cancer Res.*, **36**, 467–475.

GERONI, C., PESENTI, E., TAGLIABUE, G., BALLINARI, D., MONGELLI, N., BROGGINI, M., ERBA, E., D'INCALCI, M., SPREAFICO, F. & GRANDI, M. (1993). Establishment of L1210 leukemia cells resistant to the distamycin A derivative (FCE 24517): characterization and cross-resistance studies. *Int. J. Cancer*, **53**, 308–314.

GRANDI, M., GERONI, C. & GIULIANI, F.C. (1986). Isolation and characterization of a human colon adenocarcinoma cell line resistant to doxorubicin. Short communication. *Br. J. Cancer*, **54**, 515–518.

GROS, P., NERIAH, Y.B., CROOP, J.M. & HOUSMAN, D.E. (1986). Isolation and expression of a complementary DNA that confers multidrug resistance. *Nature*, **323**, 728–731.

KOPKA, M.L., YOON, C., GOODSELL, D., PJURA, P. & DICKERSON, R.E. (1985). The molecular origin of DNA-drug specificity in netropsin and distamycin. *Proc. Natl. Acad. Sci. USA*, **82**, 1376–1380.

MANIATIS, T., FRITSCH, E.F. & SAMBROOK, J. (1982). *Molecular Cloning. A Laboratory Manual.* Cold spring Harbor: NY.

MEADOR, J., SWEET, P., STUPECKY, M., WETZEL, M., MURRAY, S., GUPTA, S. & SLATER, L. (1987). Enhancement by cyclosporin A of daunorubicin efficacy in Ehrlich ascites carcinoma and murine hepatoma 129. *Cancer Res.*, **47**, 6216–6219.

MONTECUCCO, A., FONTANA, M., FOCHER, F., LESTINGI, M., SPADARI, S. & CIARROCCHI, G. (1991). Specific inhibition of human DNA ligase adenylation by a distamycin derivative with antitumour activity. *Nucleic Acids Res.*, **19**, 1067–1072.

PEZZONI, G., GRANDI, M., BIASOLI, G., CAPOLONGO, L., BALLINARI, D., GIULIANI, F.G., BARBIERI, B., PASTORI, A., PESENTI, E., MONGELLI, N. & SPREAFICO, F. (1991). Biological profile of FCE 24517, a novel benzoyl mustard analogue of distamycin A. *Br. J. Cancer*, **64**, 1047–1050.

RAMU, A., GLAUBIGER, D. & FUKS, Z. (1984). Reversal of acquired resistance to doxorobucin in P388 murine leukemia cells by tamoxifen and other triparanol analogues. *Cancer Res.*, **44**, 4392–4395.

ROGAN, A.M., HAMILTON, T.C., YOUNG, C.R., KLECKER, R.W. & OZOLS, R.F. (1984). Reversal of adriamycin resistance by verapamil in human ovarian cancer. *Science*, **224**, 994–996.